# Numerical proceeding to calculate impurity states in 2D semiconductor heterostructures

**DOI:** 10.1038/s41598-024-81346-6

**Published:** 2024-12-28

**Authors:** Volodymyr Akimov, Viktor Tulupenko, Roman Demediuk, Anton Tiutiunnyk, Carlos A. Duque, Alvaro L. Morales, David Laroze, Miguel Eduardo Mora-Ramos

**Affiliations:** 1 Facultad de Ciencias Básicas, Universidad de Medellín, Medellín, Colombia; 2 Grupo de Materia Condensada-UdeA, Instituto de Física, Facultad de Ciencias Exactas y Naturales, Universidad de Antioquia UdeA, Calle 70 No. 52-21, Medellín, 050010 Colombia; 3 Physics department, Donbas State Engineering Academy, Kramatorsk, Ukraine; 4 Physics department, Donbas State Pedagogical University, Sloviansk, Ukraine; 5 Departamento de Física, FACI, Universidad de Tarapacá, Casilla 7D, Arica, Chile; 6 Instituto de Alta Investigación, Universidad de Tarapacá, Casilla 7D, Arica, Chile; 7 Centro de Investigación en Ciencias-IICBA, Universidad Autónoma del Estado de Morelos, Cuernavaca, CP 62209 México

**Keywords:** Semiconductor heterostructures, Hydrogenic impurity states, Expansion method, Two-dimensional materials, Quantum mechanics, Two-dimensional materials

## Abstract

The article provides and discusses details of numerical proceeding for the expansion method to calculate energy positions and wave functions of the localized and resonant electronic states emerging in quantum well-type semiconductor nanostructures because of perturbation of confined states by the Coulomb potential of the hydrogenic impurity center. Effective mass approximation is used. Several excited both resonant and non-resonant states are calculated and classified for the case of a simple rectangular GaAs/AlGaAs quantum well. Results are compared to the ones in literature.

## Introduction

Semiconductor structures are the main source of actual technologies in the areas of sensing, computing, electronics, optical electronics and communications. Bandgap engineering is the main tool to obtain the necessary properties of semiconductor devices. Particularly, layered semiconductor structures containing quasi-two-dimensional electron gas become one of the most popular configurations for existing and perspective applications^1^. Therefore, the interest of scientific community to this kind of materials grows exponentially^2^.

Atomic impurities in semiconductor devices generally considered as an interference in an ideal crystallographic structure of semiconductor and are therefore one of the main reasons for the resistance of the whole structure. That is why modulation doping is mainly used outside the active areas as a source of free carriers. However, there are technologies that use impurities in semiconductor structures as a key feature^3^. In our previous works we proposed some ideas about possible applications of impurity-connected effects in quantum well (QW) type structures^4–7^.

The first work to calculate impurity states energies in a quantum well^8^used variational technique and gave birth to a series of subsequent publications^9–11^among others). This kind of method remains actual up to very recently^12,13^, etc.). Nevertheless, the information variational method can provide us is somewhat limited. It does not allow to reliably calculate the impurity wave function (WF), which implies that we cannot, for example, obtain matrix elements and, accordingly, the rates for all kinds of electronic transitions involving impurity states. Alternatively, expansion techniques, in which the impurity states wave function is expanded over QW subband wave functions, are used as well^14–16^. Those methods provide both wave functions and energy positions. However, they require significant processing powers and have their limitations. For instance, the well should be sufficiently deep to contain enough subbands to converge the results.

It is known that shallow impurity atom in/near the QW produces several states resembling Rydberg series. Some impurity states go out of the bandgap and appear in the background of the continuous energy spectrum of lower subbands. Such states are called resonant ones (with a reference to Fano resonance^17^) and they appear below every energy subband situated above the gap edge. Sometimes they described as broadened states.

In this work we describe in detail the numerical method based on the expansion mathematical model proposed by^14^. It can be used to numerically obtain energy positions and WFs of resonant and non-resonant impurity state in QW-like layered semiconductor heteroctructures. We use it to calculate some of the impurity states associated to a hydrogenic donor doped to the center of a rectangular GaAs/AlGaAs QW. Our results agree with an older work using the same mathematical model^15^. However, it contains much more data, which were missed by that work presumably due to the lack of access to necessary computational powers. Such data allowed us to propose the new classification of impurity states based on energy positions and the shapes of wave functions in QW-like semiconductor structures.

### Mathematical model

To apply the method below to the 2D semiconductor nanostructure first we need to find the subband electronic description of the structure without the impurity atom. To find the subband energies and wave functions *ξ*_*j*_(*z*) we must solve the one-dimensional time-independent Schrodinger equation^18^

1 $$\:{H}_{0}\xi\:\left(z\right)=E\xi\:\left(z\right)$$

For the simple case of one particle problem in a rectangular quantum well the Hamiltonian can be written as

2 $$\:{H}_{0}=-\frac{{\hbar}^{2}}{2{m}^{*}}\frac{{d}^{2}}{d{z}^{2}}+V\left(z\right).$$

Here the potential part $\:V\left(z\right)$ is a well profile $$\:V\left(z\right)=\left\{\begin{array}{c}{V}_{b},\:\:\:\:\:\:\:\:\:\left|z\right|>L/2\\\:0,\:\:\:\:\:\:\:\:\:\:\:\left|z\right|\le\:L/2\end{array}\right\}$$, *L* is a well width with *V*_*b*_ being a barrier height. In the more complex cases of external fields the Hamiltonian (2) is modified by adding respective terms. Such Hamiltonian is used when there is a small concentration of impurities or at low, usually helium, temperatures. In the case of elevated temperatures and high concentrations one can add a Hartree potential term, created by electrons and ionized impurities to the Hamiltonian and solve the Eq. (1) self-consistently along with Poisson and -maybe- electroneutrality ones. Anyway, that is a trivial task, which can be performed by available commercial^19^and free^20^ software available. For the solution we need to specify the structure geometry and some properties of the materials, particularly effective mass $\:{m}^{*}$(z). and dielectric permittivity ε(z), as function of structure growth direction coordinate z. As a result of such a solution we get al.l the necessary data for our problem, namely the wave functions of localized subbands $\:{\xi\:}_{i}\left(z\right)$, and energy positions of subband bottoms $\:{E}_{i}$.

As a base of the method to find impurity levels we used the one developed in^14^. The Hamiltonian to find the impurity states is written in cylindric coordinates *r*, *Ѳ*, *z* to take advantage of azimuthal symmetry of the system (assuming that in the directions perpendicular to z all the parameters are constant):

3 $$\:H=-\frac{{\hbar}^{2}}{2{m}^{*}}\frac{{d}^{2}}{d{z}^{2}}-\frac{{\hbar}^{2}}{2{m}^{*}}\left(\frac{{d}^{2}}{d{R}^{2}}+\frac{1}{R}\frac{d}{dR}+\frac{1}{{R}^{2}}\frac{{d}^{2}}{d{\theta\:}^{2}}\right)+V\left(z\right)-\frac{{e}^{2}}{4\pi\:\epsilon\:{\epsilon\:}_{0}\sqrt{{R}^{2}+{\left(z-{z}_{D}\right)}^{2}}}$$

where $\:{m}^{*}$ is the electron effective mass, *z*_*D*_ is z position of impurity center.

Accordingly, the WF $\Psi\left(R,\theta,z\right)$ is sought as

4 $$\:{\Psi\:}\left(R,\theta\:,z\right)\equiv\:{e}^{im\theta\:}{\psi\:}_{m}\left(R,z\right),$$

were *m* is the azimuthal quantum number and i is the imaginary unit. $\:{\psi\:}_{m}\left(R,z\right)$ is presented as an expansion over subband WFs:

5 $$\:{\psi\:}_{m}\left(R,z\right)=\sum\:_{j}{f}_{j}\left(R\right){\xi\:}_{j}\left(z\right).$$

Now using the solution of the Schrodinger equation with Hamiltonian $\:{H}_{0}$ along with the equation with $\:H$ after some math one can get the Eq. 

6 $$\:\left[\frac{{d}^{2}}{d{R}^{2}}+\frac{1}{R}\frac{d}{dR}+\left({k}_{N}^{2}-\frac{{m}^{2}}{{R}^{2}}\right)\right]{f}_{N}\left(R\right)={U}_{Nn}\left(R\right){f}_{n}\left(R\right),$$

were $\:{k}_{N}^{2}=\frac{2{m}^{*}}{{\hbar}^{2}}(E-{E}_{N})$, $\:{U}_{Nn}\left(R\right)=\frac{2{m}^{*}}{{\hbar}^{2}}\frac{1}{4\pi\:\epsilon\:{\epsilon\:}_{0}}\int\:{\xi\:}_{N}^{\text{*}}\left(z\right)\frac{{e}^{2}}{\sqrt{{R}^{2}+{\left(z-{z}_{D}\right)}^{2}}}{\xi\:}_{n}\left(z\right)dz$, and $\:{E}_{N}$ (*N* = 1,2,3…) are energy solutions of (1).

The solution of (6) is found as^14^:

7 $$\:{f}_{N}\left(R\right)=\underset{0}{\overset{{\infty\:}}{\int\:}}{G}_{N}\left(R,{R}^{{\prime\:}},E\right)\sum\:_{n}{U}_{Nn}\left({R}^{{\prime\:}}\right){f}_{n}\left({R}^{{\prime\:}}\right)R{\prime\:}dR{\prime\:},$$

where $\:{G}_{N}\left(R,{R}^{{\prime\:}},E\right)$ are Green functions given by:

8 $$\:{G}_{N}\left(R,{R}^{{\prime\:}},E\right)=\left\{\begin{array}{c}-{K}_{m}\left({k}_{N}{R}^{{\prime\:}}\right){I}_{m}\left({k}_{N}R\right),\:\:R<{R}^{{\prime\:}},\:E<{E}_{N}\\\:-{I}_{m}\left({k}_{N}{R}^{{\prime\:}}\right){K}_{m}\left({k}_{N}R\right),\:\:\:R\ge\:{R}^{{\prime\:}},\:E<{E}_{N}\\\:\frac{\pi\:}{2}{N}_{m}\left({k}_{N}{R}^{{\prime\:}}\right){J}_{m}\left({k}_{N}R\right),\:\:\:\:R<{R}^{{\prime\:}},E\ge\:{E}_{N}\:\\\:\frac{\pi\:}{2}{J}_{m}\left({k}_{N}{R}^{{\prime\:}}\right){N}_{m}\left({k}_{N}R\right),\:\:\:\:R\ge\:{R}^{{\prime\:}},E\ge\:{E}_{N}\end{array}\right.,$$

where J, N (I, K) are the Bessel functions of first (second) kind.

To solve the Eqs. (6–8) an overcomplicated version of shooting method was used in^14^. It was based on border conditions and continuity of WF expansion terms. Instead we employ a simpler way described below. Apart from this, we use the same system of equations and expect to get the same solutions within the numerical error.

Replacing in (7) integral with a sum of finite differences we get:

9 $$\:{f}_{N}\left({R}_{p}\right)=\sum\:_{q}\left[{G}_{N}\left({R}_{p},{R}_{q},E\right){R}_{q}{\varDelta\:R}_{q}\sum\:_{n}{U}_{Nn}\left({R}_{q}\right){f}_{n}\left({R}_{q}\right)\right],$$

Where $\:{R}_{p}$and $\:{R}_{q}$ get the same series of *N*_*R*_ consequent magnitudes between 0 and some $\:{R}_{max}$, $\:{\varDelta\:R}_{j}={R}_{j+1}-{R}_{j}\:(j=p,q$) $\:p,q=1..{N}_{R}$. We did not find any numerical advantage of choosing variable $\:\varDelta\:R\:$and used the simple case of uniform step $\:\varDelta\:R=const=\frac{{R}_{max}}{{N}_{R}-1}$, where $\:\:{R}_{j}=(j-1)\times\:\varDelta\:R$. For given *E*, $\:{R}_{p}$ and $\:{R}_{q}$, $\:{G}_{N}\left({R}_{p},{R}_{q},E\right)$ and $\:{U}_{Nn}\left({R}_{q}\right)$ can be calculated, and (9) in fact is a system of linear algebraic equations with respect to variables $\:f\left(R\right)$. The system consists of *N*_*s*_×*N*_*R*_ equations (*N*_*s*_ is the number of subbands used for expansion) and can be presented as:

10 $$\:M\varvec{f}=0,$$

Where ***f*** is a vector of *N*_*s*_×*N*_*R*_ elements and contains all tabulated functions *f*_*N*_, starting from 1st to $\:{N}_{s}$-th ($\:{f}_{p+\left(N-1\right)*{N}_{R}}={f}_{N}\left({R}_{p}\right),\:N=1..{N}_{s},\:p=1..{N}_{R}$), whilst *M* is a square matrix:

11 $$\:{M}_{p+\left(N-1\right)\text{*}{N}_{R},\:\:\:q+\left(n-1\right)\text{*}{N}_{R}}={\delta\:}_{pq}{\delta\:}_{nN}-{G}_{N}\left({R}_{p},{R}_{q},E\right){R}_{q}{\varDelta\:R}_{q}{U}_{Nn}\left({R}_{q}\right)$$

where $$\:N,n=1..{N}_{s},\:p,q=1..{N}_{R}$$.

In^21^ it is shown that energy positions of both resonant and non-resonant impurity levels correspond to energy values for which the condition Det(M) = 0 fulfills. So, the solution of this equation gives impurity level energies and the solution of the system (9) provides with the set of *N*_*s*_×*N*_*R*_ numerical elements of column vector $\:{f}_{j}\left(R\right)$, which allows to build the complete impurity WFs using Eqs. (4) and (5). Mathematically the equation system (9) has infinite number of solutions but owing to it is linear, we can just equate any of $\:{f}_{j}$ scalar variables to any real constant, find other variables and then normalize the WF (4) which can be written in cylindric coordinates as:

12 $$\:{{\Psi\:}}_{norm}\left(R,\theta\:,z\right)=\frac{{\Psi\:}\left(R,\theta\:,z\right)}{\sqrt{2\pi\:\underset{z=-{\infty\:}}{\overset{{\infty\:}}{\int\:}}\underset{R=0}{\overset{{\infty\:}}{\int\:}}{{\Psi\:}(\text{R},\text{z})}^{2}RdRdz}}$$

We guess that this -or similar- simple method was not proposed in^14^ because at the time the author did not have access to computers with big enough memory to properly deal with very large matrices. For instance, a typical matrix appearing in this work contained about 576,000,000 real elements.

To characterize the impurity WFs we used transverse $\:{a}_{R}$ and longitudinal $\:{a}_{z}$ localization characteristics calculated as follows.

13 $$\:{a}_{R}=\underset{V}{\overset{}{\int\:}}{{\Psi\:}}^{2}RdV=2\pi\:\underset{z=-{\infty\:}}{\overset{{\infty\:}}{\int\:}}dz\underset{R=0}{\overset{{\infty\:}}{\int\:}}{{\Psi\:}(\text{R},\text{z})}^{2}{R}^{2}dR$$

14 $$\:{a}_{z}=\underset{V}{\overset{}{\int\:}}{{\Psi\:}}^{2}RdV=2\pi\:\underset{z=-{\infty\:}}{\overset{{\infty\:}}{\int\:}}\left|{z}_{cm}-z\right|dz\underset{R=0}{\overset{{\infty\:}}{\int\:}}{{\Psi\:}(\text{R},\text{z})}^{2}RdR$$

One may interpret $\:{a}_{R}$ and $\:{a}_{z}$ as some kind of quantities equivalent to Bohr radius, defined as the most probable transverse and longitudinal distances to the center of mass of the WF $\:{z}_{cm}$. In this work we considered only the case of a center doped well $\:{z}_{D}=0$, therefore the center of the WF coincides with the well center $\:{z}_{cm}=0$ because of the well symmetry along z.

### Numerical proceeding

First, we numerically find the subband WFs $\:{\xi\:}_{j}\left(z\right)$ and respective energies $\:{E}_{j}$ as eigenfunctions and eigenvalues of Hamiltonian $\:{H}_{0}$ [Eq. (2)], employing effective mass $\:{m}^{*}$=0.067*m*_0_ (*m*_0_ is a free electron mass), dielectric constant $\:\epsilon\:$=12.565 and rectangular well profile: well width *L*=30 nm, and barrier potential *V*_*b*_=50Ry (with effective Rydberg value 1Ry=$\:\frac{{\hbar}^{2}}{2{m}^{*}{a}_{b}^{2}}=$5.80meV, $\:{a}_{b}=\frac{4\pi\:\epsilon\:{\epsilon\:}_{0}{\hbar}^{2}}{{m}^{*}{e}^{2}}$). These parameters of our structure correspond to one structure used in^15^, in order to compare the outcoming results. The task above is trivial and we shall not discuss it.

In our reference system the center of the well is at z = 0 and we take z in units of L. Here we consider impurity position in the center of the well *z*_*D*_=0.

Now we form the matrix *M* as (10) and evaluate its discriminant *D*(*E*) for a given energy *E*. To find the energy positions of impurity we must solve the equation *D*(*E*) = 0 (that provides a series of solutions described below) and then to construct the impurity wave functions for the obtained energies, we solve the Eq. 9 obtaining the corresponding functions *f*_*j*_ and form the full WFs according to Eqs. (4) and (5). Note that to correctly reproduce the WF the energy should be found with high numerical precision (we used 7 significative digits) for exactly the same set of numerical parameters.

The primary possible numerical error sources for the proceeding above are the following: (1) $\:\varDelta\:R$ in finite differences, (2) number of subbands used for expansion *N*_*s*_, (3) the maximum magnitude of *R* used to replace the infinity in the integral of Eq. 7 for the numerical purposes $\:{{R}_{max}=N}_{R}\varDelta\:R$. Our calculations showed that for all impurity states we report in this work varying parameters $\:\varDelta\:R$ and *N*_*s*_ provide good convergence of the results. Thus, for our calculations we stuck with *N*_*s*_=7 and $\:\varDelta\:R$=0.07 nm, values such that negligible changes appear when refined further. It is worth noting here that among the solutions found, the most vulnerable to an increase in ∆R turned out to be the lowest energy one, corresponding to the wave function most localized in R, which is quite expectable.

A more complex situation occurs when varying *R*_*max*_. Obviously, *R*_*max*_ should cover absolutely most of the WF, so the bigger *R*_*max*_ the better. On the other hand, we find that when *R*_*max*_ is too big in comparison to the WF extension, the calculated WF can exhibit an artificial peak at the border *R* = *R*_*max*_. The impurity level energy is not affected much by *R*_*max*_, which causes that peak, if we continue increasing *R*_*max*_, it remains stable but at some *R*_*max*_ the energy solution can disappear. We associate this behavior with an equivalent of numerical integration error. On the other hand, decreasing $\:\varDelta\:R$ does not have much effect on energy solution. So, for better reproduction of impurity WFs and to be able to find the energy of some specific impurity levels, the calculations should be performed with *R*_*max*_ within the specific reasonable range that depends on the localization of respective state.

**Fig. 1:**
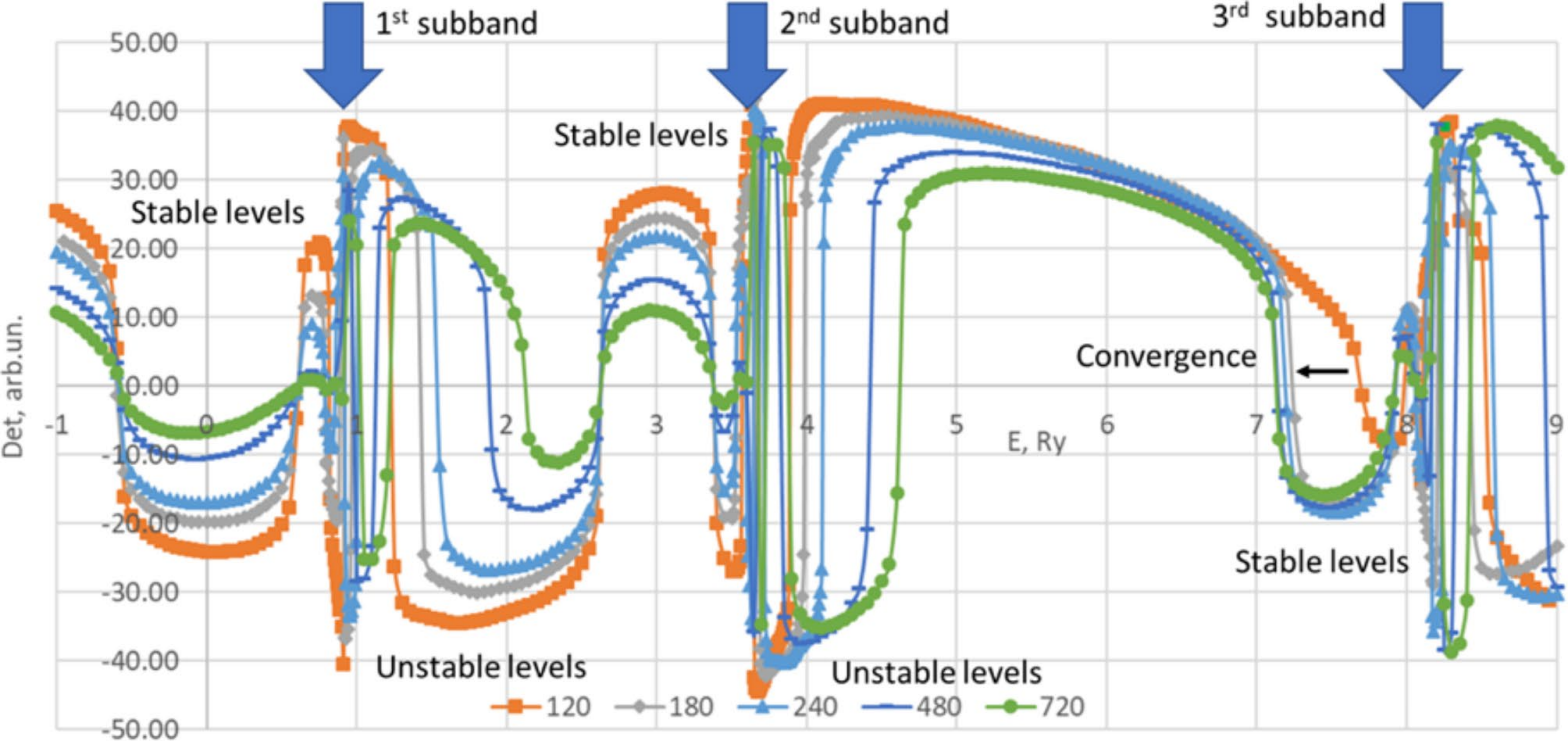
Modified determinant *D*_*M*_ of the matrix *M* for GaAs/AlGaAs 30 nm-wide rectangular quantum well with barrier height *V*_*b*_=50Ry as a function of energy in Ry for different values of *R*_*max*_. s-levels (m = 0), *R*_*max*_=120 (orange squares), 180 (grey diamonds), 240 (light blue triangles), 480 (blue minuses) and 720 nm (green circles).

The Fig. 1 is an illustration of the series of solutions provided by the method. Here the modified determinant $\:{D}_{M}$ of the matrix M is depicted as a function of energy for different *R*_*max*_. $\:{D}_{M}$ is used for visualization purposes in the graph and calculated as follows

15 $$\:{D}_{M}=\frac{\left|D\right|}{D}\times\:\left[\sqrt{{\text{l}\text{n}}^{2}\left({D}^{2}\right)+\beta\:}+\text{l}\text{n}\left({D}^{2}\right)\right],$$

where D is a matrix determinant in some arbitrary units, and β is a parameter used to adjust the visualization (here β = 100). This visualization concept has some advantages of semilogarithmic scale (particularly it conserves the smoothness and monotonousness of original determinant while allowing comparison of curve shapes with different orders of magnitude) and preserve the sign of original determinant which is important to see its zeros.

In Fig. 1 the solutions appear at the points where the curve intersects the horizontal axis. We can see that some of the solutions coincide for different *R*_*max*_ or at least converge to some energy when *R*_*max*_ grows. We shall refer to those solutions as to numerically stable ones and assume that a stable solution corresponds to a real physical impurity level. Other solutions are unstable; that is they change significantly with *R*_*max*_ and do not converge within reasonable magnitudes of *R*_*max*_. Such numerically unstable solutions may not have any physical meaning and we will ignore them. As one can see, the stable solutions tend to form a ladder asymptotically approaching the bottom of each subband whereas unstable ones are either above the subband bottoms and below the stable solutions of the next subband; or just in the immediate proximity of subband bottom. The latter can be attributed to the upper steps of respective ladders and can be calculated more exactly with bigger *R*_*max*_. All the plots in Fig. 1 are for m = 0. We performed similar calculations for m = 1 and 2 and identified stable levels.

**Fig. 2:**
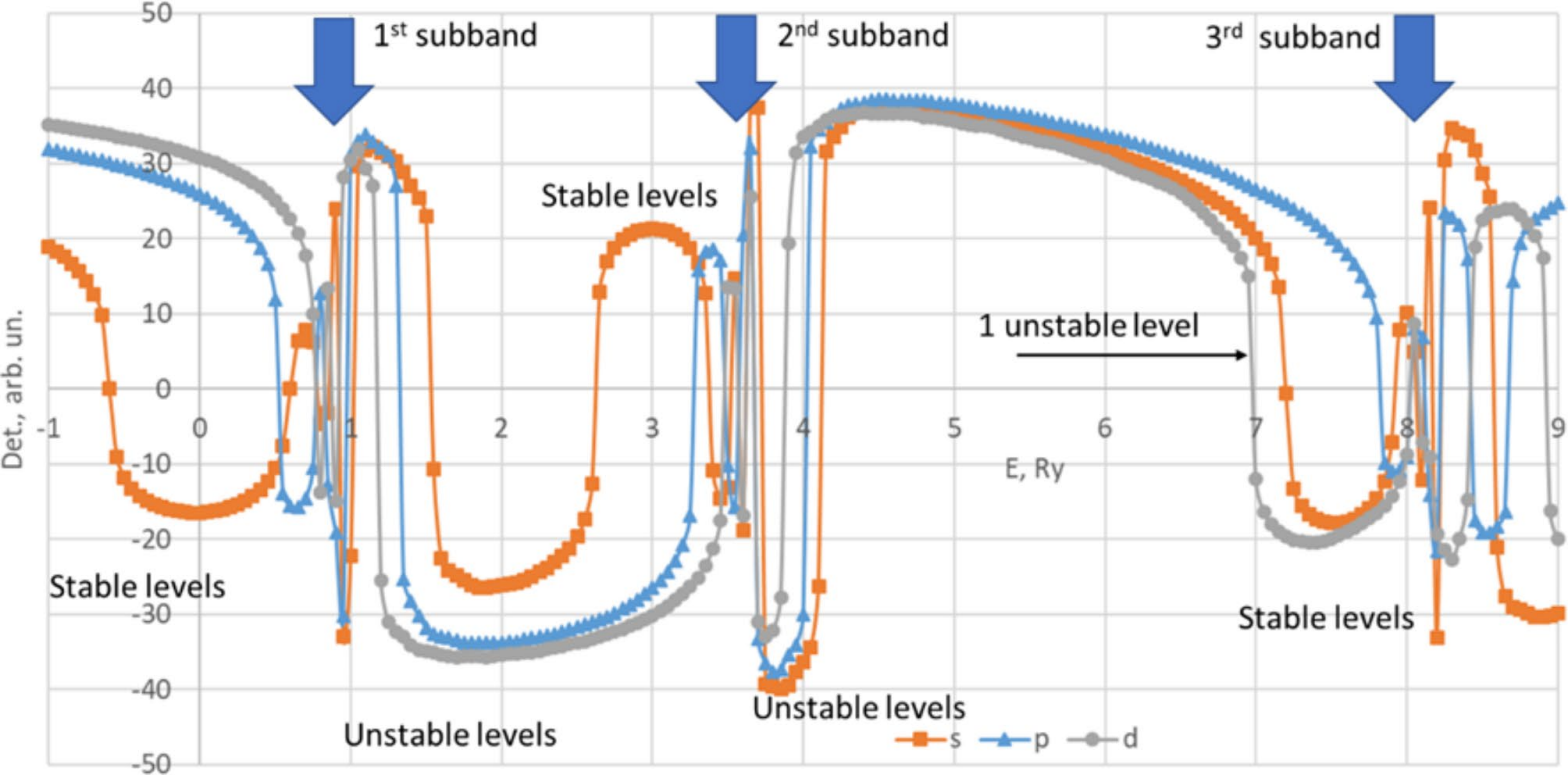
Modified determinant *D*_*M*_ GaAs/AlGaAs 30 nm-wide rectangular quantum well with barrier height *V*_*b*_=50Ry as a function of energy in Ry for different m: m=0 (s, orange squares), m=1 (p, blue triangles), m=2 (d, grey circles), *R*_*max*_=240 nm.

In the Fig. 2 we present *D*_*M*_ graphs with curves for m = 0,1 and 2 for *R*_*max*_=240 nm (which we used for calculations). The graphs for m different than 0 have the same general behavior (stable levels just below respective subbands with somewhat bigger energies for zero determinant, unstable levels above the subbands) with one notable exception: an unstable level with m = 2 just below the first stable m = 0 level (marked in the figure).

Analyzing Figs. 1 and 2 one can conclude that the set of solutions provided by zero determinants resembles a kind of Rydberg series with three independent quantum numbers. In particular, we explicitly have the azimuthal one, m, which appears in exponent of the wave function [Eq. (4)], and then determines the order of Bessel functions in Eq. (8). Two other quantum numbers can be deduced from existing solutions for each m. One of them corresponds to the number of specific subband number and another one to the step number within the respective ladder. Naturally we expect that increasing each of those quantum numbers leads to increasing energy and less localized corresponding wave function.

Here and below, for a proper classification of impurity levels we will employ the following notation. Following^15^, we use letters “s”, “p”, “d”, “f” … to refer to m = 0, 1, 2, 3… accordingly; in loose analogy to the classification of electronic states in hydrogenic atom. However, it is necessary to have in mind that the analogy is far from perfect and can be confusing in some situations. In regard, a particular impurity state is denoted as “letter, n_1_, n_2_”, where n_1_ is a subband number where 1 corresponds to the ground subband, and n_2_ is a ladder number starting from 1 for the lowest level in the ladder. For example, s11 is a ground impurity state that corresponds to −0.606Ry and s21 is a first step of the ladder below the second subband (2.622Ry), see Fig. 1.

## Discussion of physical results

**Fig. 3:**
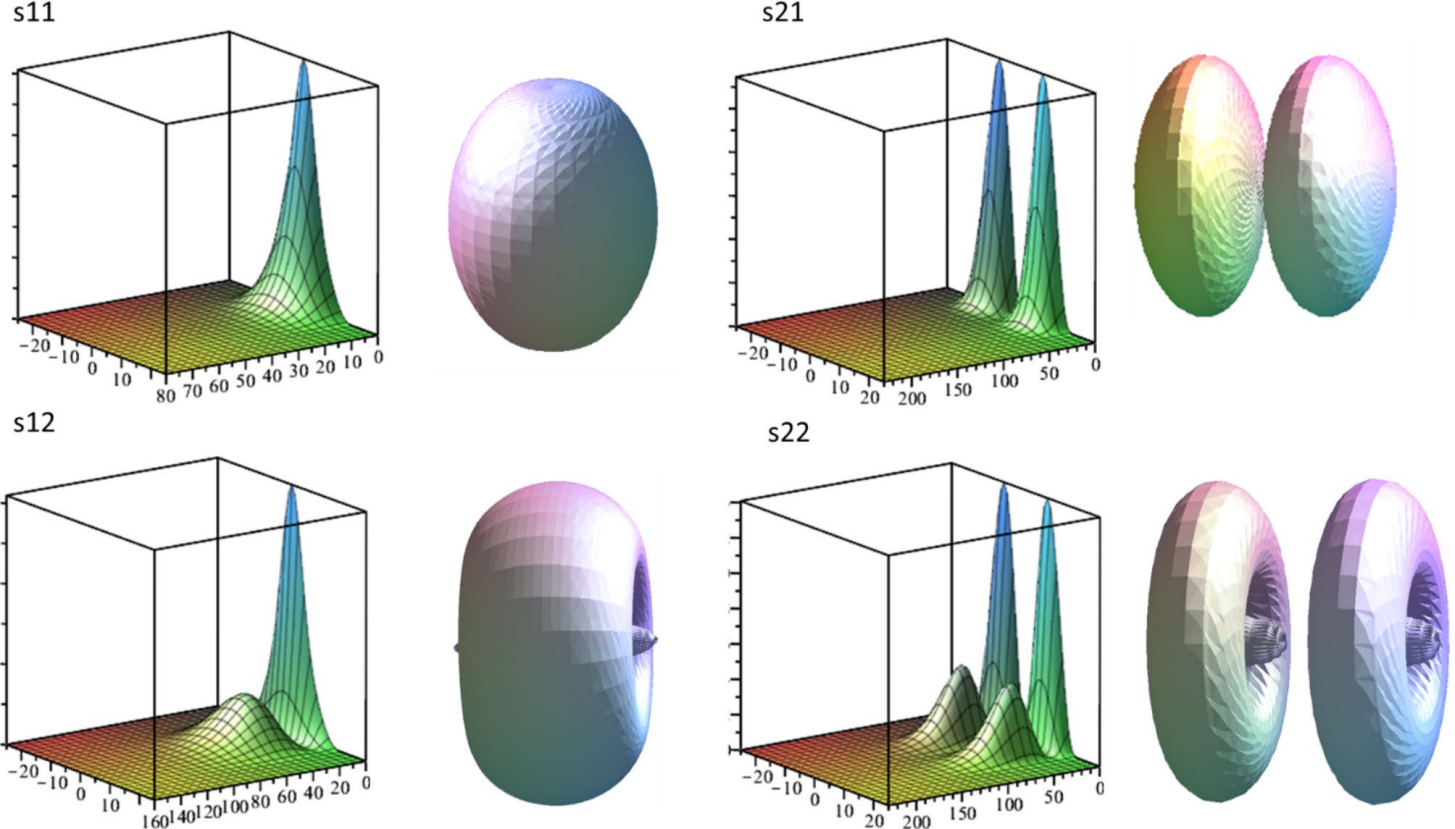
R-z plots and isosurfaces for squared wave functions of s11, s12, s21 and s22 impurity states. R and z are in nanometers.

**Fig. 4:**
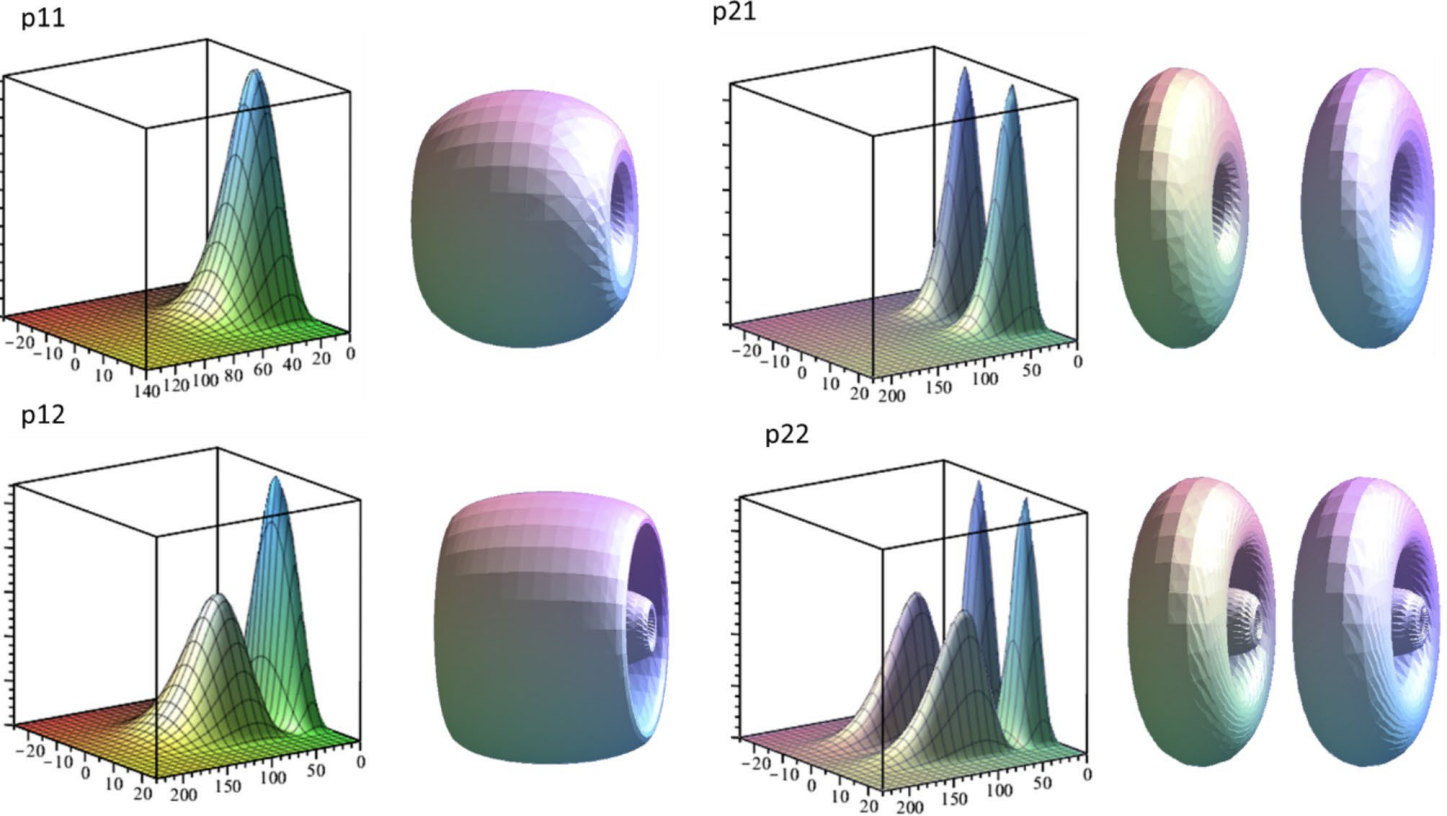
R-z plots and isosurfaces for squared wave functions of p11, p12, p21 and p22 impurity states. R and z are in nanometers.

In the Figs. 3 and 4 one can see two kinds of visual representations for the impurity wave functions of s11, s12, s21, s22 levels and p11, p12, p21, p22 levels respectively. The code of the respective state is recognized on the base of wave function shape (see discussion below, and corresponding energy positions in Table 1). The fist kind is a 3D graph against R-z plane orientated in the way that z axis corresponds the right bottom edge of the plot cube. Note that the mathematical model implies cylindrical symmetry and the only part where the angle *θ* appears is the exponent in the complete WF Eq. (4). But the squared exponent is always equal to 1, so the full squared WF is obtained by the 360° rotation of R-z graph around z axis. Another way used here to represent such state function is by means of an isosurface plot, compatible with the typical way the atomic orbitals are often represented. It is a 3D plot of surfaces that correspond to some particular value of squared wave function. The latter plot contains less information but gives better intuitive idea about volumetric shape of an electronic cloud. We can discuss the WFs in terms of peaks on R-z plots. Note that in an isosurface plot an R-z peak forms an ellipsoid-like shape or torus-like shape depending whether the peak extremum is positioned on z axis or elsewhere.

**Table 1:** Energy structure near the bottom of conduction band of 30 nm-wide GaAs/AlGaAs rectangular QW.

Level	Energy, Ry	Binding energy, Ry	*R*-localization $\:{\varvec{a}}_{\varvec{R}}$, Å	z-localization $\:{\varvec{a}}_{\varvec{z}}$, Å	Binding energy in [15], Ry
3rd subband	8.167				
s33	8.126	*0.041*	152	7.46	*0.053* (s3)
d32	8.093	0.074	145	7.83	
d31	8.016	0.151	79	7.83	
p32	8.031	0.136	106	7.19	
s32	7.921	0.246	74	7.07	
p31	7.820	0.347	67	6.74	
s31	7.198	0.969	77	5.97	
d23	3.659	−0.020	136	8.18	
p23	3.650	−0.011	154	8.18	
2nd subband	3.639				
d22	3.600	0.039	145	8.18	
s24	3.593	0.046	156	8.18	
s23	3.525	0.114	127	8.18	
p22	3.497	0.142	100	8.18	
d21	3.486	0.153	78	8.18	
s22	3.379	*0.260*	58	8.16	*0.312* (s2)
p21	3.278	0.361	36	8.16	
s21	2.622	1.017	14	8.05	
1st subband	0.911				
d13	0.896	0.015	144	4.86	
p13	0.840	0.071	153	4.85	
d12	0.834	0.077	114	4.86	
s13	0.783	0.128	116	4.85	
p12	0.761	0.150	95	4.85	
d11	0.754	0.157	76	4.86	
s12	0.600	0.311	49	4.83	
p11	0.514	*0.397*	33	4.82	*0.397* (p1)
s11	−0.606	*1.517*	10	4.42	*1.574* (s1)

**Fig. 5:**
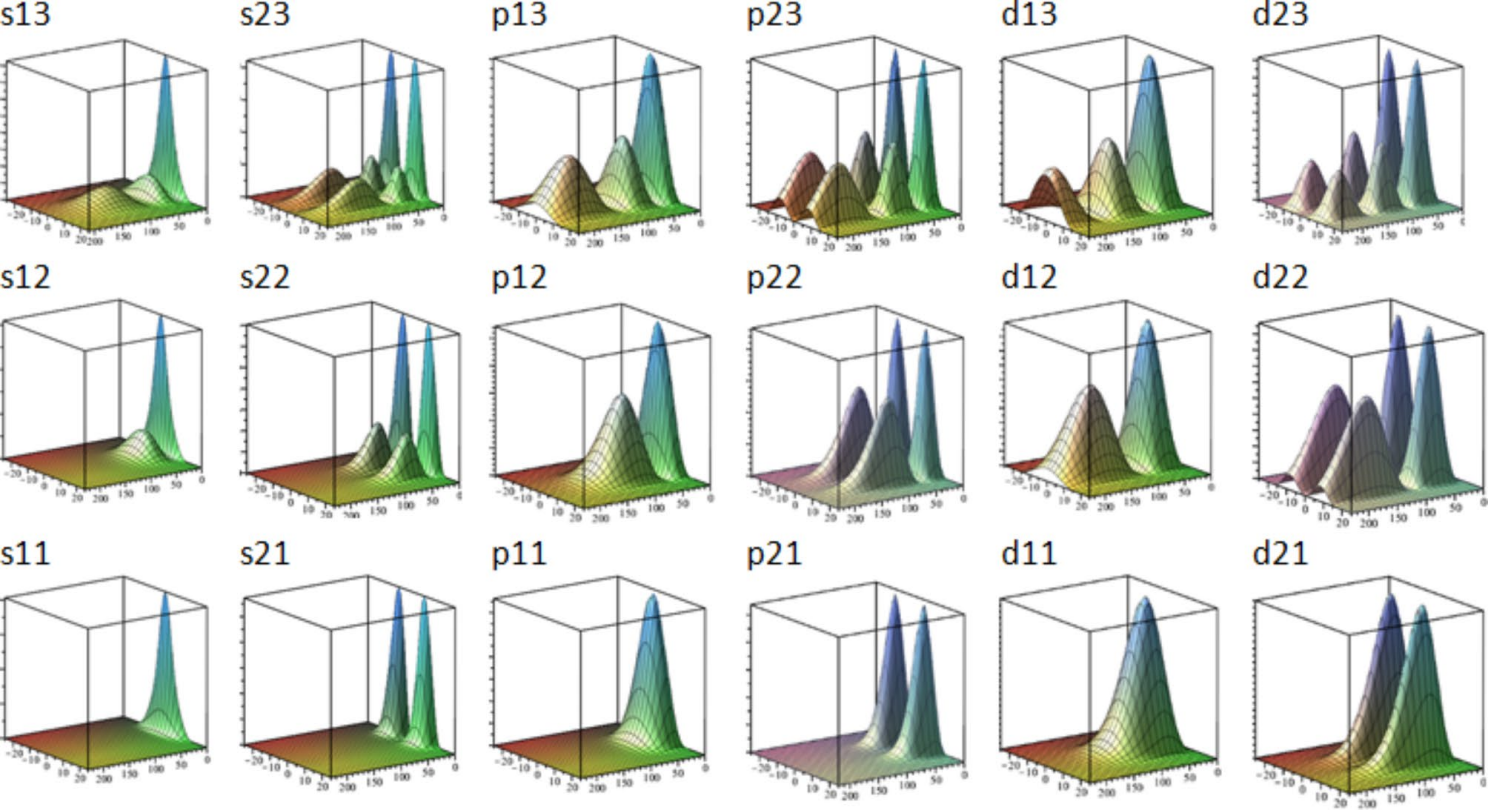
R-z plot of 18 impurity WFs, the scale in R is the same in all plots. z=−25.25 nm, *R* = 0.230 nm.

Since the impurity WFs are found through the expansion over subband states it makes sense comparing their cross-section profiles with the shapes of subband WFs. Squared subband WFs have a number of peaks equal to its assigned quantum number *j* (counted from 1). We can clearly see in Fig. 5 that the shapes of the impurity WFs follow a pattern. The peaks always form a rectangular matrix *j*×*k*. Series of levels below each subband qualitatively reproduce the shape of corresponding subband WFs in cross-sections along z so that it *j* columns of peaks along z and *k* rows along R (where *k* = 1,2,3…) are formed. And this behavior is reproduced for each *m*. Based on those observations and following to a degree the formalism of^15^ we have proposed a notation for impurity states, consisting of three indices as described above in the comments to Fig. 1 (letter, *j*, *k*). It should be noted, however, that the position in energy is secondary in our notation. In our structure, well subbands are far enough from each other in energy but in other configurations (for example, in wider QWs) the states related to different subbands can be intermixed in energy and do not form obvious ladders as in Fig. 1. Therefore, as a primary criterion for classification we take the shape of the WFs. We expect however that as soon as our indices *j* and *k* don’t have explicit presence in mathematical model, in other situation (structure asymmetry, external fields, effective mass anisotropy etc.) the identification of states by shape can be problematic though potentially possible by slowly changing parameters and following the evolution of the WF.

In all plots of Figs. 3 and 4 the units are nanometers; z is scaled from − 25 to 25 nm and the heterointerfaces between the well and the barriers correspond to −15 and 15 nm. Note that scale in R is different in distinct plots because the impurity WFs have very different localization in R (see Table 1) while this feature along z becomes mainly dictated by well barriers. Therefore, the WFs are jammed between the barriers in z and stretched in other two dimensions with $\:{a}_{R}$ growing by an order of magnitude for upper excited states. It is necessary to have in mind that for the very upper states, when the levels become numerically unstable, the $\:{a}_{R}$ is especially vulnerable to numerical error and, actually, can be significantly bigger. *s*,* p* and *d* WFs with the same j and k indices qualitatively preserve the shape with a notable exception that *s* WFs have first peak maximums exactly on z-axis while p and d peaks displace to bigger *R*.

Table 1 represents the energies of calculated impurity states. Zero energy corresponds to the well bottom potential. For all calculations, values *R*_*max*_ =*240* nm, $\:\varDelta\:R$ =*0.07* nm, *N*_*s*_=*7* were used. Note that the energies not presented in the table do not have a stable behavior with respect to *R*_*max*_ levels, which can mean that they are not localized enough within *240* nm. In Fig. 5 we present some WFs of levels not included in Table 1 because their numerical instability does not allow to reliably calculate their energies; but still it was possible to construct their wave functions for the specific *R*_*max*_.

In the first column of Table 1 we indicate each level along the lines of the code described above. In the second one we present the energy in effective Rydbergs, counted from the bottom of the well profile, which can be seen as an abscissa in graphs Figs. 1 and 2. The third column contains the values of the “binding energy” defined as the difference between the energy of corresponding subband and the impurity level. *R*-localization $\:{a}_{R}$ and *z*-localization $\:{a}_{z}$, which are calculated as described above, appear in the following two columns and, finally, in the last column we put the binding energies calculated in^15^ for the very same structure -with their notation in parenthesis. For convenience we underlined the numbers to be compared.

The states are sorted by energy in descending order. Energies above the first subband energy (that is, above 0.911 Ry) are resonant ones. We report all s, p and d (m = 0, 1 and 2) states with energies lower than the 3rd subband (including series below 1st, 2nd and 3rd subbands), which demonstrate stable energy with respect to *R*_*max*_. As expected, such states locate below the corresponding subband (the second index in our notation). Two exceptions are p23 and d23, which are above their corresponding subband and thus have negative “binding energies”. However, their difference with subband energy is minor (about 0.1 meV) and can be attributed to numerical error due to *R*_*max*_, because their WFs are strongly delocalized in *R* and, in such case, error is expected to be significant. The first (letter) and the third indices of our notation increase the energy and $\:{a}_{R}$ but apparently do it in an independent way. $\:{a}_{z}$ mainly depends on the second index and is dictated by the shape of subband wave function so that $\:{\xi\:}_{1}$, having one peak at the well center, is the most localized in *z* (*4.8* nm for a *30* nm well); antisymmetric $\:{\xi\:}_{2}$, with two peaks, is the least localized (8.2 nm), and symmetric $\:{\xi\:}_{3}$, which exhibits three peaks, tends to be somewhat less localized than $\:{\xi\:}_{2}$ because the central peak moves the WF closer to the well center, though the other peaks move away from the it, in comparison to $\:{\xi\:}_{2}$, which compensates the effect.

In general, the pattern of variation for each analyzed subband tends to be of the form “s-p-s-(p, d)-s-(p, d)” with the “binding energy” of the first s state more than 2 times bigger then the neighboring first p state.

The comparison of our results with^15^shows very good coincidence for the s11 and p11 states and somewhat a worse one for the resonant states, which however allows to identify any state confidently. The authors of^15^ revealed only one solution for *s*series below each subband, while we have found several ones. As we have already mentioned, the reason for the disagreement is, probably, their limited access to numerical processing powers, which did not allow them to scan all the energy range with proper precision. The authors of^15^ expected only one solution below each subband and probably they used a kind of bisection method to resolve the equation, which gives only one solution for an initial range. The difference in exact numeric values most probably is a result of different numerical parameters. Particularly, we believe they used a number of subbands much lesser than we did (because some of the wells they calculated contain less than 7 subbands). And naturally the upper energy levels require more refined calculation for the same precision.

## Conclusions

In our work we have calculated a series of both resonant and non-resonant hydrogenic impurity states in a 2D semiconductor heterostructure. We used an old mathematical model based on cylindric symmetry and expansion over 1D subband wave functions and improved it taking advantage of modern possibilities of computational performance. Our results refined the understanding of the pattern of impurity state series and showed its more complicated structure in comparison with what was put forward in previous works. Particularly, we have proposed a new classification system for impurity levels, based on the wave function shape, considering the wave function z-cross-section which has a qualitative resemblance to the wave function of corresponding size-quantized subband. Besides, we describe in detail the numerical proceeding along with several of its -not apparent- peculiarities, so it can be reproduced. In addition, we present and discuss the numerical outcome for a center-doped relatively narrow rectangular GaAs/AlGaAs quantum well including the shapes of the wave functions.

The results of our work can be useful for the bandgap engineering of the layered semiconductor devices using impurities to obtain necessary electrical and optical properties.

## Data Availability

The data underlying this article will be shared on reasonable request to the corresponding author.

## References

[1] Chaves, A. et al. Bandgap engineering of two-dimensional semiconductor materials. *npj 2D Mater. Appl.* **4**, 29 (2020).

[2] Xu, H., Akbari, M. K. & Zhuiykov, S. 2D Semiconductor nanomaterials and heterostructures: controlled synthesis and functional applications. *Nanoscale Res. Lett.* **16**, 94 (2021). 34032946 10.1186/s11671-021-03551-w PMC8149775

[3] Holmberg, V. C., Helps, J. R., Mkhoyan, K. A. & Norris, J. D. Imaging impurities in Semiconductor nanostructures. *Chem. Mater.* **25**, 8, 1332–1350 (2013).

[4] Tulupenko, V. et al. On the possibility of tuning the energy separation between space-quantized levels in a quantum well. *Phil Mag Lett.* **93**, 1, 42–49 (2012).

[5] Duque, C. A. et al. About possible THz modulator on the base of delta-doped QWs. *Superlattice Microst*. **87**, 5–11 (2015).

[6] Tulupenko, V. et al. Background impurities in Si0.8 Ge0.2/Si/Si0.8 Ge0.2 n-type delta-doped QW. *Phys. Stat. Sol (b)*. **254** (4), 464 (2016).

[7] Akimov, V. et al. Background impurities and a delta-doped QW. Part II: Edge doping. *Semicond. Sci. Technol.* **36**, 065011 (2021).

[8] Bastard, G. Hydrogenic impurity states in a quantum well: a simple model. *Phys. Rev. B*. **24**, 4714 (1981).

[9] Greene, R. L. & Bajaj, K. K. Energy levels of hydrogenic impurity states in GaAs-Ga1 – xAlxAs quantum well structures. *Solid State Commun.* **45**, 9, 825–829 (1983).

[10] Liu, W. & Quinn, J. J. Shallow-impurity states in semiconductor quantum-well structures. *Phys. Rev. B*. **31**, 2348 (1985). 10.1103/physrevb.31.2348 9936044

[11] Weber, G., Schulz, P. A. & Oliveira, L. E. Density of states and energy spectra of hydrogenic impurities in quantum-well wires. *Phys. Rev. B*. **38**, 2179 (1988). 10.1103/physrevb.38.2179 9946512

[12] Çakır, R. & Yıldırım, H. Binding energies of shallow donors in polar ZnO/ZnBeO quantum well. *Solid State Commun.* **379**, 115425 (2024).

[13] Liu, X. et al. Thermodynamic property of one-dimensional hydrogenic impurity in Nitride semiconductor quantum well. *Philos. Mag*. **103**, 24, 2179–2205 (2023).

[14] Vinter, B. Influence of charged impurities on Si inversion-layer electrons. *Phys. Rev. B*. **26**, 6808 (1982).

[15] Stopa, M. & DasSarma, S. Calculated shallow-donor-level binding energies in GaAs-AlxGa1 – xAs quantum wells. *Phys. Rev. B*. **40**, 12, 8466–8462 (1988). 10.1103/physrevb.40.8466 9991312

[16] Blom, A., Odnoblyudov, M. A., Yassievich, I. N. & Chao, K. A. Donor states in modulation-doped Si/SiGe heterostructures. *Phys. Rev. B*. **68**, 165338 (2003).

[17] Fano, U. Effects of Configuration Interaction on intensities and Phase shifts. *Phys. Rev.* **124**, 1866 (1961).

[18] BenDaniel, D. J. & Duke, C. B. Space-Charge effects on Electron Tunneling. *Phys. Rev.* **152**, 683 (1966).

[19] COMSOL Multiphysics^®^ v. 6.2. www.comsol.com. COMSOL AB, Stockholm, Sweden.

[20] Tan, I. H., Snider, G. L. & Hu, E. L. A self-consistent solution of Schrödinger-Poisson equations using a nonuniform mesh. *J. Appl. Phys.* **68**, 4071, 15 (1990).

[21] Abramov, A. Resonant donor states in quantum well. *Mod. Phys. Lett. B*. **25** (2), 89–96 (2011).

